# Carbon Paste Electrodes Surface-Modified with Surfactants: Principles of Surface Interactions at the Interface between Two Immiscible Liquid Phases

**DOI:** 10.3390/s23249891

**Published:** 2023-12-18

**Authors:** Ivan Švancara, Milan Sýs

**Affiliations:** Department of Analytical Chemistry, Faculty of Chemical Technology, University of Pardubice, Studentská 573, 532 10 Pardubice, Czech Republic; ivan.svancara@upce.cz

**Keywords:** carbon paste electrode, cyclic voltammetry, surfactants, surface interactions

## Abstract

Carbon paste electrodes *ex-situ* modified with different surfactants were studied using cyclic voltammetry with two model redox couples, namely hexaammineruthenium (II)/(III) and hexacyanoferrate (II)/(III), in 0.1 mol L^−1^ acetate buffer (pH 4), 0.1 mol L^−1^ phosphate buffer (pH 7), and 0.1 mol L^−1^ ammonia buffer (pH 9) at a scan rate ranging from 50 to 500 mV s^−1^. Distinct effects of pH, ionic strength, and the composition of supporting media, as well as of the amount of surfactant and its accumulation at the electrode surface, could be observed and found reflected in changes of double-layer capacitance and electrode kinetics. It has been proved that, at the two-phase interface, the presence of surfactants results in elctrostatic interactions that dominate in the transfer of model substances, possibly accompanied also by the effect of erosion at the carbon paste surface. The individual findings depend on the configurations investigated, which are also illustrated on numerous schemes of the actual microstructure at the respective electrode surface. Finally, principal observations and results are highlighted and discussed with respect to the future development and possible applications of sensors based on surfactant-modified composited electrodes.

## 1. Introduction

In general, surfactants are defined as chemical substances that are able to decrease mutual tension between two immiscible (or partially miscible) phases. This applies to the liquid–gas, liquid–liquid, and liquid–solid phase systems because all of them consist of polar and non-polar moieties [1]. Besides common functioning and use of surfactants in the form of cleaning agents [2], emulsifiers, or foaming agents [3], this particular family of chemical compounds has an unquestionable importance in the development of electrochemical sensors, which is also evidenced by myriad of the respective scientific publications [4,5,6]. Incorporation of surfactants into the configuration of various electrochemical sensors allows one to utilize highly effective interactions with the analyte that significantly increase its concentration around the sensor surface via electrostatic attraction and, at the same, minimize eventual interferences by electrostatic repulsion [7]. Also, surfactants can be widely used as additives in working media to improve the solubility of non-polar molecules in mixed or pure organic solvents [8]. Furthermore, in aqueous solutions, the molecules of surfactants form stable aggregates even above the critical micellar concentration (CMC), which can be utilized in the electrochemical sensing of some environmental pollutants [9]. Finally, the surfactant content in working media may also have a stabilization effect towards the voltammetric response of some organic analytes that tend to self-passivate after electrode reactions [10].

**Figure 1:**
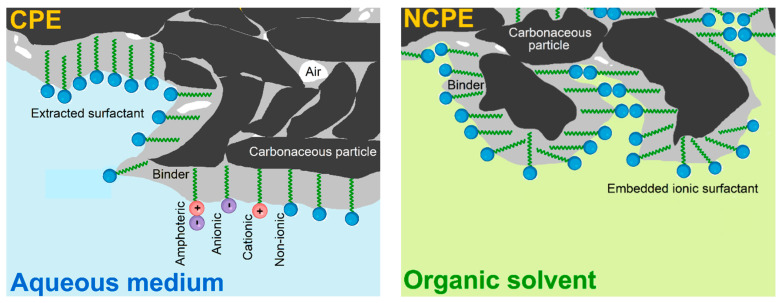
Schematic representation of the microstructure of two types of surfactant-modified CPEs.

Regarding carbon paste electrodes (CPEs) in combination with surfactants, the resultant configurations represent one of the most popular types of chemically modified variants of these heterogeneous electrochemical sensors, whose electrode material is usually prepared by hand-mixing powdered graphite with non-polar and highly viscous liquid binders (typically, mineral or silicone oils [11,12]). In fact, there are two basic types of surfactant-modified CPEs, namely (i) surface-modified [13,14] and (ii) bulk-modified variants [15,16]. The difference between these two types can be well illustrated in their specific microstructure as shown in Figure 1.

In the first variant, the surface of the bare CPE is covered by an adsorbed layer of the molecules of a surfactant, when its hydrophobic tails (“water-hating” moiety) are firmly anchored via extraction into the non-polar binder whereas the hydrophilic heads (“water-loving” part) protrude into the surrounding solution [7,13]. These surface-modified CPEs are stable only in purely aqueous solutions unlike the second variant, bulk-modified CPEs, which are resistant against exposure to organic solvents. Therefore, such configurations had been designated by their inventor Adams as “nonaqueous CPEs” (NCPE [15]) and characterised by the presence of a rather high content of ionic surfactant in the solid state admixed mechanically into the native carbon paste during its preparation [16].

So far, only a few reports have been devoted to an explanation of why the intentional treatment by surfactants causes a several-fold increase in the current response and a shift of the peak potential to lower values. Besides the already mentioned electrostatic interactions [7], Kauffmann et al. pointed out the removal of a thin layer of the binder from carbon particles by the action of surfactant molecules and called it “carbon paste erosion” [13], causing a hydrophilization and significant increase in the electrochemically active surface area (ECSA). Moreover, the different wettability of the bare carbon paste layer of a non-polar nature in contrast with a polar surface of surfactant-modified carbon paste can be another interpretation of significantly altered properties of modified CPEs [17].

Thus, it seems that all the above-mentioned phenomena somehow participate in the resultant properties of surfactant-modified CPEs and the working conditions used. And this means that the type and amount of the surfactant attached, length of its tail, pH value, ionic strength, and the composition of the supporting electrolyte co-determine which of these phenomena would prevail or even dominate.

And this was exactly what motivated us to elaborate a new systematic study—to our knowledge, the first one with such a focus and intention. Our experiments were arranged and realised as a voltammetric investigation with two model redox couples: hexaammineruthenium (II)/(III) as a cation system and hexacyanoferrate (II)/(III) representing the anionic pair. In addition to this, the surfactants selected for this study were typical compounds from this family of specifically behaving compounds, when our choice reflected also their main types. Therefore, it can be assumed, for example, that all cationic surfactants extracted into the pasting liquid of CPE will to a greater or lesser extent electrostatically attract large anions (potential analytes) from the solution, subsequently form the corresponding ionic pairs, and at the same time repel equally charged ions (potential interferences). It is believed that the observations and results obtained from these measurements could be useful for the future development of specific voltammetric methods utilizing heterogenous sensors with incorporated surfactants.

## 2. Materials and Methods

### 2.1. Chemicals and Reagents

Hexaammineruthenium(III) chloride (98%), potassium hexacyanoferrate(III) (≥99.0%), didecyldimethylammonium bromide (DDAB, 98%), cetyltrimethylammonium bromide (CTAB, ≥99%), sodium dodecyl sulfate (SDS, ≥90%), sodium dodecylbenzenesulfonate (SDBS, technical grade), and Triton™ X-100 (analytical grade) were purchased from Merck KGaA (Darmstadt, Germany). Other chemicals were obtained from Lach-Ner (Neratovice, Czech Republic). Ultrapure water with resistivity ≥18.3 MΩ cm was prepared by a Milli-Q^®^ deionization unit (Merck Millipore, Burlington, Massachusetts, USA).

### 2.2. Apparatus

Each voltammetric measurement was performed in a typified glass vessel containing 10 mL of 0.1 mol L^−1^ acetate (pH 4), phosphate (pH 7), or ammonia buffer (pH 9); all serving as a working medium when modeling mild acidic, neutral, and basic conditions. The three-electrode cell consisted of the working bare (or modified) CPE, a reference Ag/AgCl/3 mol L^−1^ KCl from Metrohm (Herisau, Switzerland), and a platinum sheet as the auxiliary electrode (Elektrochemické detektory, Turnov, Czech Republic). The electrode cell was connected to a potentiostat/galvanostat (model PGSTAT101, Autolab) operated through NOVA 1.11 software, both from the already presented Metrohm. The pH of the respective supporting electrolyte(s) was checked by a conventional pH meter with a combinatory glass electrode calibrated with three commercial buffers.

### 2.3. Preparation of Carbon Paste Electrodes Modified with Surfactant

Native (unmodified) graphite-based pastes with purposely varied content of mineral oil (10, 20, and 30% *w*/*w*) were prepared by thorough hand-mixing with the appropriate amount of carbon powder using a ceramic pestle and mortar. The resultant homogenized carbon pastes were firmly embedded into cavities of Teflon^®^ piston-driven electrode holders [18] with an end-hole forming a surface diameter of 3 mm. Before any surfactant accumulation, the surface of the resultant CPE assemblies was always manually renewed by extruding of small portion of carbon paste and smoothing it with a wet filter paper.

**Figure 2:**
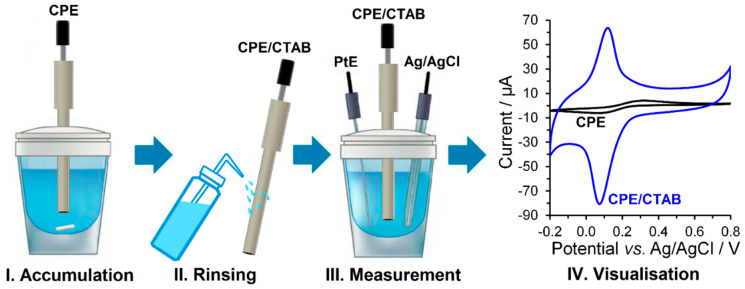
Individual steps of *ex-situ* modified CPEs with surfactant preparation.

The modification of the surface with a surfactant was carried out by immersing the bare CPE into an aqueous solution of the given surfactant (0, 0.1, 0.5, 1.0, and 2 mmol L^−1^) stirred at 400 rpm for chosen time (0, 2, 4, 6, 8, and 10 min). After rinsing with a stream of distilled water, the surfactant-modified CPEs could be taken for their subsequent electrochemical characterisation. All the necessary steps, including the final visualisation of the respective voltammogram, are shown in Figure 2.

### 2.4. Procedures

All the voltametric measurements were performed in the linear sweep voltammetric mode (LSV) in three different buffers as 0.1 mol L^−1^ aqueous solutions (pH 4, 7, and 9) over two potential ranges (from 0 to +2 or from 0 to −2 V vs. Ag/AgCl/3 mol L^−1^ KCl) at a scan rate of 50 mV s^−1^ and a potential step of 5 mV to define the corresponding residual currents and potential limits. Furthermore, cyclic voltammetry (CV) was employed as the technique of choice and a diagnostic tool for the investigation of reaction kinetics, where the respective buffer had been spiked with 500 μmol L^−1^ [Ru(NH_3_)_6_]Cl_3_ or K_3_[Fe(CN)_6_] and measured from −0.2 to +0.8 V, at a scan rate varying from 50 to 500 mV s^−1^.

## 3. Results and Discussion

### 3.1. Electrode Stability

As known from the previous studies and measurements [19], CPEs prepared from traditional liquid binders and modified with the accumulated surfactants are mechanically stable only in purely aqueous solutions and aqueous–organic mixtures, where the content of polar organic solvent may not exceed a ratio of 10% (*v*/*v*). As expected for this type of modification, the use of polar liquid binders [17] is not very recommended because the presence of a surfactant in the accumulation medium may dissolve them, which inevitably leads to the disintegration of the electrode material—the carbon paste itself.

Knowledge about the stability of the current signal measured over time is quintessential for intended electroanalytical purposes, for which the accumulation of the analyte on the electrode surface via ion-pairing is mainly utilized [20]. Basically, it can be assumed that ion-pairing of cationic surfactants with [Fe(CN)_6_]^3−^ anion or anionic surfactants with [Ru(NH_3_)_6_]^3+^ cation is time dependent. The whole process can be accelerated by a higher concentration of counterions in the working medium and by stirring, which both represent another two aspects for more effective formation of ion pairs, together with the molecular diffusion and further enhancement by electrostatic interactions.

**Figure 3:**
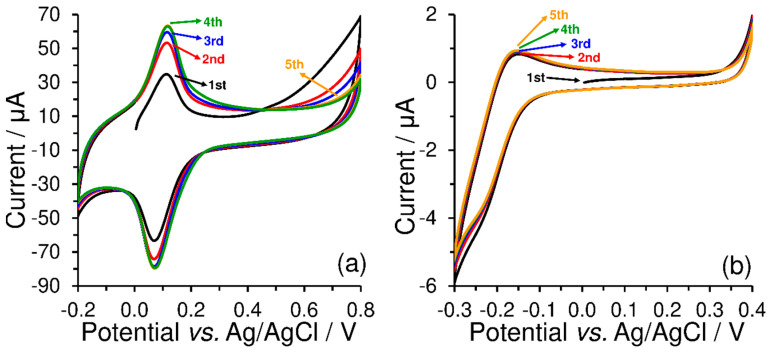
Repetitive cyclic voltammograms (five cycles) of 500 μmol L^−1^ K_3_[Fe(CN)_6_] obtained at CPE-CTAB (**a**) and 500 μmol L^−1^ K_3_[Ru(NH_3_)_6_] obtained at CPE-SDS (**b**), both obtained by measurements in 0.1 mol L^−1^ phosphate buffer (pH 7) at 50 mV s^−1^.

As shown in Figure 3, redox peak current responses ($I_{p}^{a}$ and $I_{p}^{c}$) increased simultaneously with the number of cycles, when constant current responses of the used redox couples were observed only after the third repetition. From the comparison of the current yields of the respective electrode reactions performed in 0.1 mol L^−1^ phosphate buffer (pH 7), it is evident that electrostatic interactions between the sulfonate groups and [Ru(NH_3_)_6_]^3+^ cations are not similar to those of the quaternary ammonium salts with the [Fe(CN)_6_]^3−^.

Such an observation just confirms the fact that the accumulation step should be optimised in detail within the development of stripping voltammetric methods based on ion-pair formation because the efficiency and repeatability of accumulation can be significantly affected by the sample matrix, especially by the occurrence of accompanying ions. In contrast, the cleaning step requires only the extrusion of the carbon paste, polishing the surface with a wet filter paper, and formation of the new layer of surfactant.

### 3.2. Potential Range and Effect of pH

Regarding the accessible potential window in the cathodic region and certain limitations for CPEs [12], it has been confirmed that the same drawback is also the case of surfactant-modified CPEs. The reason is that, in all configurations of carbon paste-based electrodes and sensors, it is not possible to remove the molecular dioxygen entrapped inside the carbon paste mixture and encapsulated in the graphite used, as well as penetrating in during manual mixing. In any case, when a CPE is polarized in the negative direction, the captured oxygen gives rise to a large reduction signal that deforms a wide part of the cathodic potential region. Due to this unavoidable phenomenon, the cathodic polarizability of surfactant-modified CPEs has not been studied.

**Figure 4:**
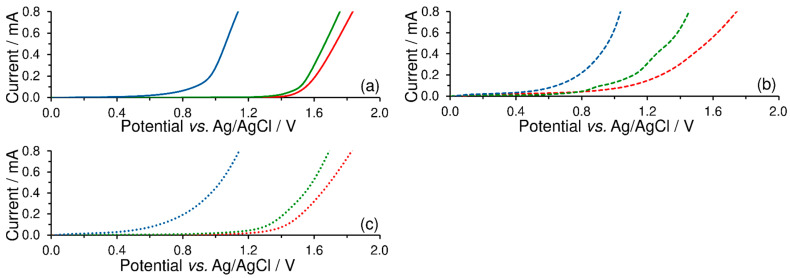
Linear-sweep voltammograms of 0.1 mol L^−1^ acetate buffer (pH 4; red), phosphate buffer (pH 7; green), and ammonia buffer (pH 10; blue curve) recorded on bare CPE (solid; **a**), CPE-CTAB (dashed; **b**), and CPE-SDS (dotted lines; **c**) at potential step of 5 mV and scan rate of 50 mV s^−1^.

To investigate the effect of a surfactant upon the anodic potential range, LSV of blank buffers was performed first. Here, it was confirmed that the width of the anodic window is given by electrochemical oxygen evolution, which depends mainly upon the pH value of the working medium selected as shown in Figure 4a. Regarding the effects of surfactants, Figure 4b illustrates that the presence of cationic surfactant attached to the CPE surface significantly shortens the potential range in neutral and acidic media. On the other hand, the use of an anionic surfactant as a surface modifier slightly widens the anodic overpotential, reducing a bit of the baseline currents, as is evident from Figure 4c.

### 3.3. Double-Layer Capacitance

**Figure 5:**
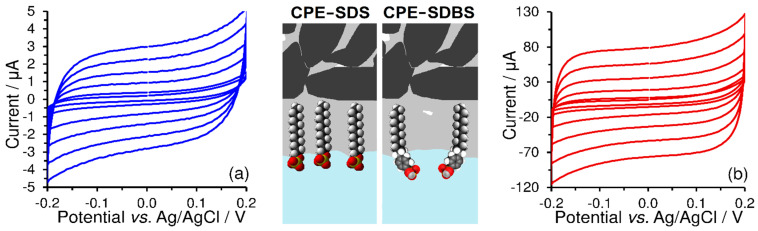

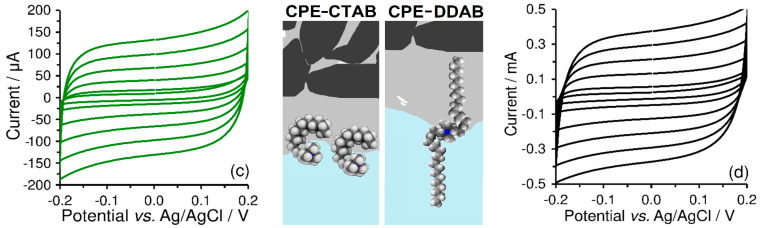
Cyclic voltammograms of 1 mol L^−1^ KOH obtained at CPE-SDS (**a**), CPE-SDBS (**b**), CPE-CTAB (**c**), and CPE-DDAB (**d**) at potential step of 5 mV and scan rates of 50, 100, 200, 300, 400, and 500 mV s^−1^. Scheme of surfactant molecules extracted into pasting liquid of CPE.

If CPEs are modified with surfactants that differ only in the type of polar head (i.e., sulfonate, benzenesulfonate, cetyltrimethylammonium, and lauryl dimethylammonium) and then investigated by CV in 0.1 mol L^−1^ KOH at a scan rate ranging from 50 to 500 mV s^−1^ (see Figure 5), one obtains completely different values of double-layer capacitance ($C_{DL}).$ Namely, the respective values were calculated as follows: 0.068 mF cm^−2^ for the bare CPE, 0.088 mF cm^−2^ for CPE-SDS, 2.7 mF cm^−2^ for CPE-SDBS, 3.9 mF cm^−2^ for CPE-CTAB, and, finally, 11.1 mF cm^−2^ for CPE-DDAB. Thus, it seems that the obtained $C_{DL}$ values largely reflect the surface microstructures of modified CPEs. For example, compared to SDS, the presence of a benzene ring in the molecular structure of SDBS extends the length of the non-polar tail, which gives rise to a longer distance between the electrode surface and the ions in the Helmholtz double layer. Analogically, this is a reason why a non-polar long-alkyl chain of DDAB markedly increased the double-layer capacitance in comparison with that at three methyl groups of CTAB. Such interpretation implies that the choice and number of accumulated surfactants increase the background currents, which may deteriorate the analytical performance of the corresponding voltammetric methods.

The final values of the double-layer capacitance are influenced not only by the molecular structure of the surfactants used, but also by their amount present on the CPE surface and the concentration of supporting electrolyte in the working medium. It follows that the double-layer capacitance can also be considered as a measure of accumulated surfactants because it is increased with a higher content of accumulated surfactant up to a constant value that reflects the extraction balance of surfactant between two non-mixable liquids (non-polar pasting liquid and aqueous solution).

### 3.4. Type of Surfactant and the Effect of Ionic Strength

**Figure 6:**
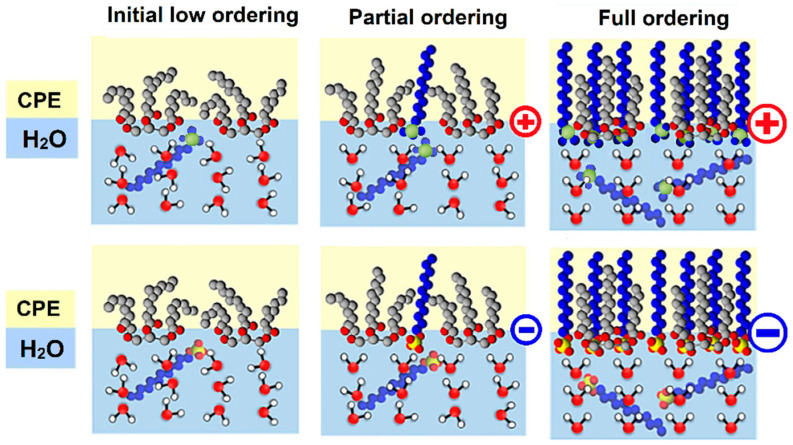
Representative illustration of accumulation of surfactants (via extractive anchoring of the molecules of CTAB and SDS into triacylglycerides). Blue and yellow colour represent an aqueous phase (working medium) and non-aqueous phase of triacylglycerides (paste binder), respectively.

As shown in Figure 6, the selection of surfactant type significantly affects the polarity of electrostatic charge, while the final amount of accumulated surfactant, therefore, and also the overall charge, depends upon the surfactant concentration, accumulation time, and intensity of stirring. Within optimisation of *ex-situ* modification, it was found that the accumulation from an aqueous solution of 1 mmol L^−1^ CTAB, continuously stirred at 400 rpm for 10 min was sufficient for complete surface coverage of a CPE with the content of mineral oil in the range of 10–30% (*w*/*w*). As expected, the use of higher concentrations of surfactants only shortened the accumulation time, whereas higher values of stirring speed had no significant effect on the electrochemical behaviour of resultant modified CPEs.

**Figure 7:**
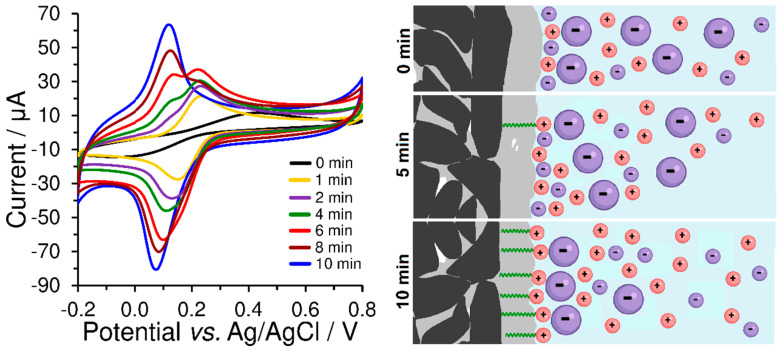
Cyclic voltammograms of 500 μmol L^−1^ K_3_[Fe(CN)_6_] obtained at bare CPE (black curve) a differently prepared CPE-CTAB electrodes in 0.1 mol L^−1^ phosphate buffer (pH 7) at 50 mV s^−1^. These modified CPEs differed only in the accumulation time of the surfactant from its 1 mmol L^−1^ aqueous solution at a stirring speed of 400 rpm and room temperature. The corresponding scheme illustratively shows the different CPE coverage depending on the accumulation time of CTAB and the ion-paired [Fe(CN)_6_]^3−^ anions (large violet particle).

As evident from Figure 7, the peak current response of the redox marker seems to more suitable measure of the final amount of accumulated surfactant than the previously mentioned double-layer capacitance. Moreover, the position and shape of the obtained voltammetric peaks reflect the coverage of the carbon paste electrode by the surfactant layer because the presence of surfactant significantly affects the diffusion of bulky [Fe(CN)_6_]^3−^ anions. In the case of using bare CPE, smaller and more mobile electrolyte ions form a Helmholtz layer, while [Fe(CN)_6_]^3−^ anions are present in the diffuse layer. However, if molecules of CTAB surfactant are present on the electrode surface, the Helmholtz layer will also be formed by their polar (positively charged) heads which will cause the binding of negatively charged [Fe(CN)_6_]^3−^ anions and obtaining almost axisymmetric peaks, typical for substances in the adsorbed state. This is the main reason why the surface of the CPE is not completely covered with the CTAB surfactant layer, and why it is possible to obtain pairs of anodic peaks, even though a one-electron reaction has occurred. Thus, the first anodic peak at a lower potential corresponds to the ion-paired [Fe(CN)_6_]^3−^ anion, while the second one corresponds to the [Fe(CN)_6_]^3−^ anion in the diffuse layer.

From a practical point of view, this typical behaviour of hexacyanoferrate (II)/(III) redox marker could be used for checking the sufficient coverage of heterogeneous carbon electrodes and serve in the optimisation of their preparation conditions, such as surfactant concentration, accumulation time, stirring speed, and temperature, which influences the rate of reaching the surfactant extraction equilibrium between the polar aqueous phase and the non-polar CPE binder. If the dependence of the height of the peak on the surfactant accumulation time is plotted on the graph, a typical saturation curve (extraction isotherm) will be obtained, from which the time required to achieve maximum coverage of the electrode surface can be determined. This equilibrium period can be shortened by increasing the surfactant concentration and stirring speed during accumulation, whereas setting speed values higher than 400 rpm did not cause any improvement, and therefore this value can be considered to be optimum.

For an open-circle arrangement (i.e., potential-free circuit), it can be assumed that there will be only electrostatic interactions between the modified carbon paste surface and the analyte. Thus, attractive or repulsive forces between the charged particles will strengthen or weaken the diffusion transfer of the analyte towards the electrode surface. Here, it should be noted that this would be valid only for conditions at a low ionic strength of the working medium, when the Helmholtz-like double layer is formed from the positively charged quaternary ammonium or negatively charged sulfonate, respectively, and the oppositely charged ions in the solution, when this structure dominates, accompanied by the free K^+^ and [Fe(CN)_6_]^3−^ ions scattered around in the working medium. In this case, one can conclude that [Fe(CN)_6_]^3−^ species forms the ion pairs with quaternary ammonium salts, which is reflected in the shape of almost symmetrical opposite voltammetric peaks that resemble voltammograms of substances in the adsorbed state; see Figure 7 and compare the corresponding CVs presented therein.

**Figure 8:**
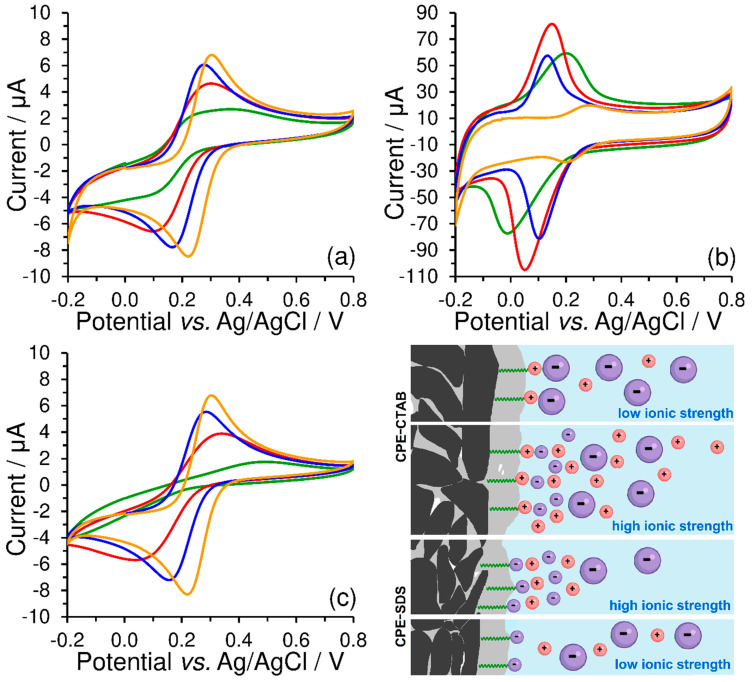
Cyclic voltammograms of 500 μmol L^−1^ K_3_[Fe(CN)_6_] recorded at bare CPE (**a**), CPE-CTAB (**b**), and CPE-SDS (**c**) in 1 (orange), 0.1 (blue), 0.05 (red), and 0.01 mol L^−1^ KCl (green curve) at 50 mV s^−1^. Below and right: scheme of electrostatic interaction between the extracted molecules of surfactant and the free ions in working medium, where the large particles with negative charge (violet colour) represent the [Fe(CN)_6_]^3−^ anion.

Unlike the peak separation, Δ*E*_p_ = 85 mV, obtained at the bare CPE, a value close to 25 mV was achieved using CPE-CTAB for hexacyanoferrate (II)/(III) redox couple in 0.01 mol L^−1^ KCl at a scan rate of 50 mV s^−1^. However, if the concentration of the supporting electrolyte is higher than 1 mol L^−1^ (see orange curves in Figure 8), the effect of the electrostatic interaction will be already less pronounced, manifested solely via the increased background (baseline). This behaviour was also confirmed by the experiments performed with CPE-SDS, when electrostatic repulsion had decreased the reversibility of the redox system investigated (compare green curves in Figure 8a,c), while almost identical cyclic voltammogram as at the bare CPE could be obtained at high ionic strength (see again orange curves in Figure 8a,c). For illustration, all the above-mentioned phenomena are shown by the scheme in Figure 8.

From these findings, it can be concluded that the ionic strength can fundamentally affect the reversibility of the investigated redox couples under certain conditions (low values of the polarization rate), which can be confirmed by comparing the calculated values of peak separation (Δ*E*_p_) and peak current ratio (|*I*_p_^a^/*I*_p_^c^|), as shown in Table 1. For example, the maximum separation of 543 mV and the peak current ratio of 7.97 clearly demonstrate the effect of electrostatic repulsion between the negatively charged SDS surfactant heads and [Fe(CN)_6_]^3−^ ions in a working medium containing 0.01 mol L^−1^ KCl.

As already mentioned, the rate of polarization must also be taken into consideration because the formation of the electrode double layer is time dependent, with more mobile Cl^−^ anions reaching the electrode surface faster than larger and slower [Fe(CN)_6_]^3−^ anions. This is also the reason why the electrostatic interactions cannot be well observed at higher values of the scan rate.

### 3.5. Ion-Pair Formation in-between the Anionic and Cationic Surfactants

**Figure 9:**
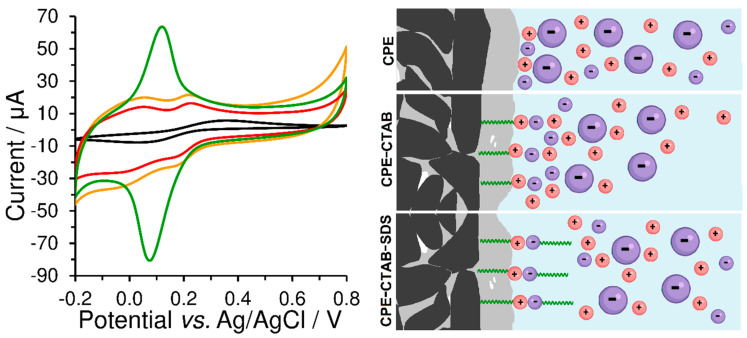
(**Left**) Cyclic voltammograms of 500 μmol L^−1^ K_3_[Fe(CN)_6_] recorded at the bare CPE (black curve), CPE-CTAB (green), CPE-CTAB-SDS (orange line), and CPE-Triton X-100 (red) in 0.1 mol L^−1^ phosphate buffer (pH 7) at 50 mV s^−1^. (**Right**) Scheme of electrostatic interaction between the extracted molecules of surfactant and free ions in the working medium, where large negatively charged particles represent the [Fe(CN)_6_]^3−^ anion.

Ion pairing of cationic surfactants with anionic ones is generally known and practically used for their titration with potentiometric indication, which enables us to determine their total contents [21]. Here, a two-stage accumulation performed *ex-situ* was used to modify the CPE surface with a double layer of mutually paired surfactants. At first, the bare CPE was immersed into a stirred solution of 1 mmol L^−1^ CTAB for 10 min and so-obtained CPE-CTAB configuration then transferred into a stirred solution of 1 mmol L^−1^ SDS for 10 min (or vice versa). The resultant CPE modified with two types of surfactants in parallel, i.e., the CPE-CTAB-SDS type, exhibited a completely non-polar character, which was comparable with that represented by CPE surface modified with a non-ionic Triton X-100 (CPE-Triton X-100 type); see Figure 9. The latter was prepared by immersing the bare CPE into a stirred solution with 1 mmol L^−1^ Triton X-100 for 2 min. From Figure 8, it can be deduced that the surface modifications of CPE with mutually paired surfactants or with non-ionic surfactants are not very suitable for voltammetric analysis of substances of ionic nature because of the undesirable increase in the background currents.

### 3.6. Electrochemical Activity of the Surfactants Studied

As discussed in the previous sections, the choice of surfactant, its amount at the CPE surface, and the composition of the working medium represent essential conditions for achieving optimal current yields of the electrode reactions with respect to sensitivity and selectivity in electroanalytical applications. Electrostatically controlled diffusion of substances and their subsequent ion pairing can significantly affect the transport towards and backwards from the electrode interface [22].

Within a study on the effect of scan rate with 0.5 mol L^−1^ K_3_[Fe(CN)_6_] in 0.1 mol L^−1^ phosphate buffer (pH 7) at the bare CPE, CPE-CTAB, and CPE-SDS, the slopes of 0.42, 0.96, and 0.37 μA mV^−1^ s were obtained from the corresponding dependences of log $I_{p}^{a}$ and log $I_{p}^{c}$ vs. scan rate (*ν*). Such values suggest that there is adsorption-controlled redox transformation of [Fe(CN)_6_]^3−/4−^ at CPEs modified with cationic surfactants, in contrast to the same electrode reaction at the bare CPE exhibiting a diffusion-controlled process that can be weakened by electrostatic repulsion at CPEs modified with anionic surfactants. Concerning the individual combinations with electrochemically active cations, one can anticipate more-or-less opposite relations.

Thus, it can be concluded from the above-described observations that usually recommended calculation of the electrochemically active surface area (ECSA) using the Randles–Sevcik equation (RSE [23]) cannot be applied herein, because the spontaneous diffusion of model substances is being affected by specific interactions with the active functional groups of the surfactant(s).

If the RSE is to be used for the ion-paired substances, a larger ECSA would always be obtained, which could result in a false indication—an erosion of the carbon paste surface layer ([13] with exposure of “naked” carbon particles). On the other side, the phenomenon of carbon paste erosion cannot be excluded from consideration when CPEs with more polar pasting liquids are used, and, in these configurations, both processes may take place in parallel.

Using the Nicholson method [24], heterogeneous electron-transfer rate constant ($k^{0}$) of 0.9 × 10^−3^, 1.6 × 10^−3^, and 8.97 × 10^−3^ cm s^−1^ for ferro/ferricyanide redox couple could be determined at the bare CPE, CPE-SDS, and CPE-CTAB, respectively. Such values confirm previous statements about higher electrochemical activity of surfactant-modified CPEs than the unmodified ones. In the case of sufficiently high ionic strength, even an anionic surfactant can positively affect the electrode kinetics of so-modified CPE. Namely, these factors lead to a more polar character of the surfactant-modified carbon paste surface, which is reflected, among others, in a nearly twice-faster electron transfer.

## 4. Conclusions

In this article, we have explored the electrochemical properties of traditional carbon paste electrodes in special configurations when their surfaces have been modified with different types of surfactants. Our results and observations revealed that the choice of a modifier, its amount, and the composition of the working medium are capable of principally affecting the transport of a substance to the surface of CPEs modified by surfactants, when the resultant mechanism of transport involves electrostatically driven diffusion and the subsequent ion pairing with possible participation of the erosion effect, thereby an evident increase in the electron transfer can be achieved.

In prospect, our future activities will be focused on further investigations of the individual findings reported above because we are convinced that new and more detailed results could contribute to the further development of electrodes and sensors modified with surfactants and their new possibilities in electroanalysis. In particular, the finding that the ion-pair formation may lead to an extraordinary increase in the sensitivity of such configurations (see, e.g., [25] and the detection of Ag^+^ ions down to the picomolar level), but it can also be beneficial for methods requiring pre-analysis operations, such as sampling when either positively or negatively charged, as well as completely uncharged molecules can be effectively (pre)accumulated at the surfactant-modified electrodes and sensors.

## Figures and Tables

**Table 1:** Effect of supporting electrolyte concentration on electrochemical reversibility of hexacyanoferrate (II)/(III) redox couple.

	CPE		CPE-CTAB		CPE-SDS	
*c* (KCl)/mol L^−1^	Δ*E*_p_/mV	|*I*_p_^a^/*I*_p_^c^|	Δ*E*_p_/mV	|*I*_p_^a^/*I*_p_^c^|	Δ*E*_p_/mV	|*I*_p_^a^/*I*_p_^c^|
0.01	273	1.66	201	0.84	543	7.97
0.05	197	1.17	106	0.83	272	1.26
0.1	91	1.07	25	0.73	96	1.08
1	85	1.05	81	1.11	85	1.05

Notes: Data were calculated from values obtained from cyclic voltammetric measurements of 500 μmol L^−1^ K_3_[Fe(CN)_6_] in different KCl solutions at bare CPE and CPEs surface modified with CTAB or SDS at 50 mV s^−1^. *Ex-situ* modification was performed by immersing the bare CPE into continuously stirred 1 mmol L^−1^ aqueous surfactant solution at 400 rpm for 10 min.

## Data Availability

All the relevant data are only provided in the present paper.
